# Modeling diarrhea in children under five in Somaliland: A machine learning analysis using SLDHS 2020 data

**DOI:** 10.1371/journal.pone.0345482

**Published:** 2026-03-25

**Authors:** Yahye Hassan Muse, Mukhtar Abdi Hassan, Abdisalam Hassan Muse, Hibak Ismail, Saralees Nadarajah, Hodo Abdikarim

**Affiliations:** 1 Faculty of Science and Humanities, School of Postgraduate Studies and Research (SPGSR), Amoud University, Borama, Somalia; 2 Department of Mathematics, University of Manchester, Manchester, United Kingdom; Gadjah Mada University Faculty of Medicine, Public Health, and Nursing: Universitas Gadjah Mada Fakultas Kedokteran Kesehatan Masyarakat dan Keperawatan, INDONESIA

## Abstract

**Background:**

Diarrhea remains a leading cause of morbidity and mortality among children under five years of age, particularly in low- and middle-income countries. This study investigated the prevalence and determinants of diarrhea in Somaliland using nationally representative data from the 2020 Somaliland Health and Demographic Survey (SLDHS) 2020.

**Methods:**

We employed a cross-sectional study design and analyzed data from 1,112 women (aged 15–49) and their children under the age of five from six geographic regions in Somaliland. Variables were selected based on data availability in SLDHS 2020, including socioeconomic, demographic, and environmental factors. We employed descriptive statistics and binary logistic regression to identify significant associations of the variables with childhood diarrhea. Additionally, supervised machine-learning models (Logistic Regression, Probit Regression, Random Forest, Decision Tree, and SVM) were used to identify key determinants of diarrhea.

**Results:**

The overall prevalence of diarrhea was 7.2%, with significant regional variation (Togdheer: 12.5%; Awdal: 4.24%). Nomadic households had a significantly higher incidence (8.62%) than rural (2.41%) and urban (5.16%) households. Logistic regression analysis highlighted region, household wealth index, and sanitation access as significant predictors. Interestingly, maternal educational level was not significantly associated with the prevalence of diarrhea. The Decision Tree model achieved the highest accuracy (92.3%) and sensitivity (33.3%), while Logistic Regression had specificity >97%.

**Conclusion:**

This study underscores the importance of region-specific public health strategies focused on improving access to water and sanitation, especially in nomadic and low-income populations. Despite the high overall accuracy, the machine-learning models indicated that the predictive accuracy for positive diarrhea cases could be further refined. Efforts to alleviate diarrhea among young children in Somaliland should prioritize the enhancement of infrastructure and sanitation resources in underserved communities.

## 1. Background

Diarrhea is defined as the passage of loose or watery stools three or more times in 24 hours.most commonly caused by infectious agents such as bacteria, viruses, and parasites [1]. Infectious diarrhea can be contracted in several ways, including through direct oral contact, food- or waterborne infections, person-to-person contact, or fecal-oral transmission. Diarrheal infections usually spread in an unsanitary environment with an overabundance of flies, trash, and an inadequate water supply [2].

Globally, Diarrhea is a significant public health concern and remains one of the leading causes of morbidity and mortality among children. Diarrhea is the sixth most common cause of death worldwide and the third most common cause of morbidity in people of all ages, causing over one in five child deaths annually, or roughly 1.5 million, and is the second most common cause of death in children under five years worldwide. It kills more young infants altogether than measles, malaria, and acquired immune deficiency syndrome (AIDS) combined [3].

Diarrhea is the third leading cause of death among children aged 1–59 months and is both preventable and treatable. Annually, it claims approximately 443,832 lives of children under five, as well as 50,851 children aged 5–9 years. Many of these cases could be avoided through access to safe drinking water, proper sanitation, and good hygiene practices. Globally, childhood diarrhea accounts for nearly 1.7 billion cases each year, and it remains a major contributor to malnutrition among children under five [4]

Children from low- and middle-income nations account for the majority of these deaths [5]. Diarrheal sickness is estimated to account for 9% of all deaths among children under the age of five worldwide, according to a 2021 report from UNICEF. Despite the availability of straightforward, efficient therapy, this indicates that over 1,200 young children pass away every day, or over 440,000 children annually [6]

Children under the age of three in low-income nations often experience three episodes of diarrhea annually. The child loses the nourishment required for growth during every episode. As a result, diarrhea turns into a major contributor to malnutrition, with undernourished children having a higher risk of developing diarrhea [7]. However, because morbidity from diarrheal diseases has remained mostly stable over the past 20 years, comparatively few patients in wealthy countries die from diarrhea.

In Ethiopia, access to safe piped water is often limited and costly. Diarrhea is the leading cause of illness, causing nearly half of all deaths among young children. Childhood diarrhea affects between 13.5% and 30.5% of children, highlighting a significant health issue [8,9]. To stop diarrhea, action is required. This action includes providing safe drinking water, using better sanitation techniques, and washing hands with soap. An oral rehydration solution (ORS), a mixture of clean water, sugar, and salt, can also be used to treat diarrhea. Furthermore, a 10-to 14-day additional course of treatment consisting of 20 mg zinc tablets reduces the duration of diarrheal illness and enhances results [7].

A range of environmental, behavioral, and socio-demographic factors contribute to the occurrence of diarrhea. Inadequate of access to safe drinking water, poor sanitation, and insufficient hygiene are central drivers [10]. Over 280 million children under the age of five live in households without access to better sanitation facilities, and over 125 million children under the age of five live in households without an improved source of drinking water [11]. About 88% of diarrheal disease deaths, or more than 1.5 million deaths in children under five annually, occur in underdeveloped countries due to a combination of factors, including contaminated drinking water, a lack of access to sanitation, and inadequate water availability for hygiene [11].

The under-five mortality rate in low-income nations was 73 deaths per 1000 live births, about 14 times higher than the average rate in high-income countries (i.e., 5.3 deaths per 1000 live births), according to the World Health Organization (WHO) 2016 [12]. In sub-Saharan Africa, the overall prevalence of diarrhea is 15.3%. Compared to their peers, children of mothers who were 15–24 and 25–34 years old, as well as those whose mothers had only a primary education or no education, were more likely to experience diarrhea. years, and those with secondary education had an increased risk of diarrhea. children from the poorest, poorer, and middle class [13].

Research has indicated that maternal education can affect mothers’ attitudes towards childhood diarrhea and lead to greater changes in the health of their children. Additional research shows that there is no direct correlation between maternal qualities, such as money and education and diarrhea in children; instead, additional factors, including residency disparities, modify the link [14]. Child health outcomes can be explained by a variety of factors, including household and maternal characteristics, such as bottle feeding, the number of children in the home, the mother’s age and education, as well as the availability of clean drinking water and sanitary facilities. Numerous studies have shown that specific demographic factors are associated with the occurrence of diarrhea. For instance, regardless of water and sanitation conditions, children with more educated mothers typically have a lower prevalence of diarrhea, which is attributable to a greater understanding of good hygiene [15].

Breastfeeding exclusively during the first 4–6 months of life protects against diarrheal illnesses [16–20]. A study conducted in Ethiopia found that 8.2% of mothers and 18.0% of children under five years of age experienced diarrhea [21]. There was a strong correlation between the age of the infant, the duration of breastfeeding, the mother’s education, the availability of a latrine facility, and the mother’s history of recent diarrhea [21].Childhood diarrhea is more likely to be influenced by the mother’s place of residence and the child’s age. A mother’s higher education level was linked to a lower frequency of diarrhea in children. Size of the home and the availability of drinking water were two aspects of the household that statistically significantly influenced the prevalence of diarrhea in children. Diarrhea is highly correlated with not having a toilet, not treating water at home, not properly disposing of baby waste, and not having better water sources [22].

The risk of diarrhea was correlated with the mother’s age, the child’s size at delivery, the mother’s area, sex, and employment level. In the context of the WASH environment, the likelihood of diarrhea was raised by waiting 30 minutes or more for water, using outdated restroom facilities, placing such facilities in yards or other public spaces, and not having a place to wash your hands with soap and water [23]. Diarrhea was significantly predicted in children aged 7–11 months if they were the second born, had not had a rotavirus vaccination, and were fed by hand [24].

Extremely low sanitation standards and the population’s extensive use of contaminated water have had a massive impact on the health landscape in Somaliland, where diarrheal illnesses have been prevalent and serious issues since 1994, with yearly epidemics. These contributing variables are more noticeable in IDP camps, where living conditions are significantly more difficult, and they operate as hubs for cholera outbreaks that are endemic [25]. Poor sanitation facilities in IDP camps increase the risk of waterborne illnesses and provide an ideal environment for the spread of diarrheal infections. Residents who frequently experience displacement as a result of environmental or armed conflicts deal with inadequate access to clean water, cramped living circumstances, and poor hygiene facilities, all of which increase their risk of contracting diarrheal diseases.

This study introduced the application of machine learning (ML) methods to analyze the prevalence and determinants of diarrhea among children under five in Somaliland, offering advantages over traditional statistical approaches. Unlike conventional regression models, which assume linear relationships and may overlook complex interactions, ML techniques effectively capture non-linear patterns and high-dimensional data structures. By utilizing algorithms such as decision trees, random forests, and support vector machines, this study improved predictive accuracy and identified hidden risk factors. The flexibility and robustness of ML models enhance the reliability of data-driven public health interventions, providing deeper insights for policy development and targeted disease prevention strategies.

[26] In Somaliland, diarrhea affected 4% of children under the age of five, and 2% of those children sought treatment or guidance from a healthcare facility. Better sanitation practices and availability of clean drinking water, particularly in homes, would stop the spread of diseases such as dysentery and diarrhea throughout the nation. Water that is piped into a household, yard, or plot is available to 17% of households [27].

To date, there have been no studies on the causes of diarrhea in children under five in the country, but only two studies have been conducted in the IDPs of Hargeisa. Using nationally representative data, this study investigated the prevalence and contributing variables of diarrhea in children under five years of age in Somaliland.

Against this backdrop, this study introduced the application of machine learning (ML) methods to analyze the prevalence and determinants of diarrhea among children under five in Somaliland, offering advantages over traditional statistical approaches. Unlike conventional regression models, which assume linear relationships and may overlook complex interactions, ML techniques can effectively capture non-linear patterns and high-dimensional data structures. By employing algorithms such as decision trees, random forests, and support vector machines, this study enhanced predictive accuracy and identified hidden risk factors, thereby improving the reliability of data-driven public health interventions and providing deeper insights for policy development and targeted prevention strategies. Using nationally representative data, the study investigated the prevalence and determinants of diarrhea among children under five in Somaliland, addressing a critical evidence gap: previous research had largely been confined to IDP camps in Hargeisa and did not capture the broader socio-demographic and environmental diversity of the country. Specifically, this study aimed to identify key risk factors—including maternal education, household water access, sanitation conditions, and hygiene practices—associated with childhood diarrhea, and to generate empirical evidence to inform targeted public health interventions and policy recommendations to reduce the burden of diarrheal disease and improve child health outcomes in Somaliland.

## 2. Methods

### 2.1. Study area

This study utilized data from Somaliland, a self-declared state in the Horn of Africa, situated between Djibouti, Ethiopia, and Somalia, and bordering the Gulf of Aden. Somaliland covers approximately 176,119 km² and comprises six administrative regions used in this analysis: Awdal, Maroodi-Jeex, Sahil, Togdheer, Sanaag, and Sool.

### 2.2. Study design and study period of health demographic survey

The Somaliland Health Demographic Survey (SLHDS), which was conducted in 2019 and published as the Somaliland Demographic Health Service (SLDHS) for 2020, was conducted using a cross-sectional design.

### 2.3. Sample size and sampling of health and demographic survey

Data from 1,112 women aged 15–49 years, who were included in the SLHDS data collection, were used in this study. Sampling considered six geographic regions for stratification, in addition to residency (rural, urban, and nomadic). Geographic Information System (GIS) software was used to select the enumeration area (EA) for both urban and rural areas. A total of 2,806 residential structures (1869 in urban regions and 937 in rural areas) were included in the sampling frame. Based on the size of residential structures, 35 enumeration areas (EAs) were selected using a probability-based method. Ten primary sampling units (PSU) were selected from the 35 EAs using the probability proportional sampling technique after the households in the EAs were listed. For those who were itinerant, 24 temporary nomadic settlements (TNS) were built, keeping a model frame in mind.

The list of TNS was used as a sampling frame, and the expected number of households in each TNS served as a measure of size. A total of 1,448 TNS residential structures were discovered, and the EAs of both urban and rural residents were selected in the same manner. The last sampling unit, households, was selected using systematic sampling.

### 2.4. Variables

#### 2.4.1. Dependent variable.

This paper focuses on diarrheal disease among children under five in Somaliland. The dependent or outcome variable was directly captured in the DHS data as Diarrhea was defined as three or more loose/liquid stools within 24 hours in the two weeks preceding the survey.

#### 2.4.2. Independent variables.

Numerous factors have been linked in previous research to diarrheal illness in children under five [13,24,28–32] This study divided the correlations of diarrheal illness in children under five into two groups, including individual level and community-level variables including maternal age group, maternal education level, child twin, age of the child, household has a television, household has a radio, ever used internet, wealth index combined and number of household members. The study then provides variables pertaining to community-level factors, such as residence, region, time to get water, toilet facility and source of drinking water.

The selection of independent variables for this study was guided by a comprehensive review of the existing literature on the determinants of childhood diarrhea, as well as a conceptual framework that considers individual-, household-, and community-level factors. In addition to the availability of data within the SLDHS 2020 dataset, priority was given to variables with demonstrated associations with diarrhea in prior research, including maternal characteristics (age and education), child-specific attributes (age and twin status), household factors (wealth index, sanitation access, and water source), and Community-level factors (e.g., water access, sanitation infrastructure) were included as they reflect shared environmental risks per WHO guidelines. The selection process also considered the potential for variables to capture the underlying mechanisms related to hygiene practices, ecological exposures, and socioeconomic vulnerabilities, ensuring the inclusion of variables with both theoretical relevance and practical significance for informing targeted public health interventions in Somaliland.

### 2.5. Data source

The data evaluated in this study were obtained from the Somaliland Health and Demographic Survey (SLHDS). During the survey, skilled interviewers gathered data from both urban and rural areas by using the CSPro Android platform. Information was provided by 30 houses in each of the ten enumeration regions within each geographic stratum. Similarly, in the nomadic areas, data were collected from 30 randomly selected households in each enumeration district. One day before data collection in each TNS, the list of residences was reviewed to ensure accuracy and completeness.

The microdata used in this analysis are publicly available from the Somaliland microdata repository: https://microdata.nbs.gov.so/index.php/catalog/50

### 2.6. Data quality assurance

Before collecting the survey data, the data collectors completed a pretest and received training. GPS tracking of field operations was utilized to assist with georeferencing so that geolocated data could be collected and data gathering was continuously monitored.

### 2.7. Data processing and analysis

Before the analysis, data cleaning procedures were implemented to address missing values in the Somaliland Demographic and Health Survey (SLDHS) dataset. Participants with missing values for the outcome variable (diarrhea status) were excluded to ensure the integrity of the dependent variable. For the independent variables, a careful review of the frequency and pattern of missingness was conducted. Variables with >15% missing values (e.g., maternal occupation) were excluded. Missingness was entirely assumed at random (MCAR), and mode imputation addressed <5% missingness. A single imputation method was employed for the remaining variables with minimal missing data. Specifically, mode imputation was used for categorical variables, replacing missing values with the most frequently occurring category within each variable. These data-cleaning steps ensured a consistent and comprehensive dataset for robust analysis.

The data was cleaned after removal from the SLHDS. Before the analysis, participants for whom no outcome variables were available in the datasets were excluded. STATA version 17 was used to import and analyze the data. Calculations were performed using descriptive statistics, including frequency and percentage. Bivariate and multivariable binary logistic regression were used to analyze the components that are related to diarrheal disease among children under five with a P-value less than 0.05 in order to determine the factors associated with diarrheal disease among children under five.

### 2.8. Machine learning models

In this study, we employed several supervised machine learning models to predict the determinants of diarrhea in children under five. Below are the models used, along with their definitions and respective formulas.

1 **Logistic Regression**

Logistic Regression (LR) is a statistical model used to solve binary classification problems. It predicts the probability that a given input point belongs to a particular category by applying a logistic function to a linear combination of the input features. The output is constrained between 0 and 1, making it suitable for scenarios where the outcome is binary (e.g., success/failure, yes/no) [33]. The formula for the logistic regression is:


$$P(Y=\text{1}|X)=\frac{\text{1}}{\text{1}+e^{-\left(\beta_{\text{0}}+\beta_{\text{1}}X_{\text{1}}+\beta_{\text{2}}X_{\text{2}}+\cdots +\beta_{n}X_{n}\right)}}$$

where P(Y = 1|X) is the probability of the event occurring (e.g., a child having diarrhea), $\beta_{\text{0}}$ is the intercept, $\beta_{i}$ represents the coefficients for each predictor variable $X_{i}$ and e is the base of the natural logarithm

2 **Probit Regression**

Probit Regression is like Logistic Regression but uses the cumulative distribution function of the standard normal distribution to model the probability of a binary outcome. This is particularly useful when the assumption of a logistic distribution is inappropriate. Probit models are often employed in situations where the dependent variable is binary, and the underlying distribution of the error terms is assumed to be normally distribute al [34].

The probability of an outcome in the probit regression is given by:


$$P(Y=\text{1}|X)=\Phi \left(\beta_{\text{0}}+\beta_{\text{1}}X_{\text{1}}+\beta_{\text{2}}X_{\text{2}}+\cdots +\beta_{n}X_{n}\right)$$

where Φ represents the cumulative distribution function of the standard normal distribution, β_0_ is the intercept, and β_i_ is the coefficient for each predictor X_*i*_.

3 **Decision Tree**

A Decision Tree is a flowchart-like structure used for classification and regression tasks. It splits the data into subsets based on the value of the input features, creating branches that lead to decision and leaf nodes, which represent the final output or classification. The tree is constructed by recursively partitioning the data based on feature values that yield the highest information gain or the most significant reduction in impurity [35]. The information gain for a split *S* on feature X is defined as


$$Information\;Gain(S,X)=H(S)-\;\sum {\mathrm{\backslash nolimits}}_{i=\text{1}}^{m}\frac{\left|Si\right|}{\left|S\right|}\text{ H}(\text{Si})$$

where H(S)is the entropy of the dataset before the split, S*i* are the subsets after the split, and |Si|/|S| is the proportion of data in each subset.

4 **Random Forest**

Random Forest is an ensemble learning method that constructs multiple Decision Trees during training and outputs the mode of predictions for classification tasks, or the average for regression tasks. This approach enhances the accuracy and robustness of the model by reducing overfitting, which is a common problem in single Decision Trees. Random Forests leverage the concept of bagging (bootstrap aggregating) to create diverse trees from random subsets of the data, thereby improving predictive performance [36,37].

Random Forest is an ensemble learning model that builds multiple decision trees and averages their predictions to improve accuracy and reduce overfitting. Each tree was trained on a random subset of data, and the final prediction was made by averaging the results (for regression) or taking a majority vote (for classification).


$$\hat{Y}=\frac{\text{1}}{T}\sum {\mathrm{\backslash nolimits}}_{t=\text{1}}^{T}ft(X)$$

where T is the number of trees, ft(X) represents the prediction of the t-th tree, and $\hat{Y}\;$is the final prediction.

5 **Support Vector Machine (SVM)**

The SVM is a supervised learning model used for classification and regression tasks. It works by determining the hyperplane that best separates the data points of different classes in a high-dimensional space. The SVM aims to maximize the margin between the closest points of the classes, known as support vectors. This model is particularly effective in high-dimensional spaces and is robust against overfitting, especially in cases where the number of dimensions exceeds the number of samples [38]. An SVM is a classifier that finds the hyperplane that maximizes the margin between two classes. For a binary classification problem, the decision boundary is represented as


f(x)=sign(w. X+b)

where w is the weight vector, X is the feature vector, and b is a bias term. The sign function determines the class based on the side of the hyperplane where X falls.

These models were selected for their diverse strengths in handling classification tasks, allowing us to evaluate and compare predictive capabilities in the context of identifying factors contributing to diarrhea among children under five years of age in Somaliland.

### 2.9. Model Training and Evaluation

To optimize the performance and ensure the generalizability of the machine learning models, a stratified 5-fold cross-validation approach was employed on the 80% training dataset. This involved partitioning the training data into five equal folds, ensuring that each fold maintained a representative distribution of the outcome variable (diarrhea status). For each model, the hyperparameters were tuned using a grid search technique within the cross-validation loop. Specific hyperparameters tuned included the number of trees and maximum tree depth for Random Forest, kernel type (e.g., linear, radial basis function), regularization parameter for Support Vector Machines, and regularization strength for Logistic and Probit Regressions. The optimal hyperparameter combination was selected based on the average performance across all folds as measured by the area under the receiver operating characteristic curve (AUC-ROC). This rigorous cross-validation and hyperparameter tuning process aimed to prevent overfitting and ensure reliable performance on unseen data, thereby enhancing the robustness and reproducibility of the study findings.

## 3. Results and findings

### 3.1. Descriptive statistics

As depicted in Table 1. This analysis leverages data from the 2020 Somaliland Demographic and Health Survey (SLDHS) to explore the prevalence and determinants of diarrhea among children under five years of age in Somaliland. This investigation employed both univariate statistics and bivariate chi-square tests to examine the correlations between diarrhea incidence and a range of demographic, socioeconomic, and household factors. Identifying these key contributing factors provides valuable insights into targeted public health interventions, particularly in regions with limited access to healthcare.

**Table 1:** Univariate and Bivariate analysis of diarrhea prevalence among children under five in Somaliland (SLDHS 2020).

Variable	Levels	Frequency (%)	Diarrhea recently		P-Value
			Yes (%)	No (%)	
Mothers age in five year group	15-19	156 (14.03)	13 (8.33)	143 (91.67)	0.818
	20-24	500 (44.96)	35 (7.00)	465 (93.00)	
	25-29	336 (30.22)	21 (6.25)	315 (93.75)	
	30-34	94 (8.45)	4 (4.26)	90 (95.74)	
	35-39	22 (1.98)	2 (9.09)	20 (90.91)	
	40-44	4 (0.36)	0 (0.00)	4 (100.0)	
Region	Awdal	118 (10.61)	5 (4.24)	113 (95.76)	**<0.001**
	Woqooyi/galbeed	191 (17.18)	7 (3.66)	184 (96.34)	
	Togdheer	264 (23.74)	33 (12.50)	231 (87.50)	
	Sool	266 (23.92)	10 (3.76)	256 (96.24)	
	Sanaag	273 (24.55)	20 (7.33)	253 (92.6)	
Residence	Rural	249 (22.39)	6 (2.41)	243 (97.59)	**0.002**
	Urban	155 (13.94)	8 (5.16)	147 (94.84)	
	Nomadic	708 (63.67)	61 (8.62)	647 (91.38)	
Mothers level of education	No education	820 (73.74)	55 (6.71)	765 (93.29)	0.677
	Primary	192 (17.27)	11 (5.73)	181 (94.27)	
	Secondary	64 (5.76)	5 (7.81)	59 (92.19)	
	Higher	36 (3.24)	4 (11.11)	32 (88.89)	
Household has a radio	Yes	98 (8.81)	4 (4.08)	94 (95.92)	0.271
	No	1,014 (91.19)	71 (7.00)	943 (93.00)	
Household has a television	Yes	38 (3.42)	1 (2.63)	37 (97.37)	0.304
	No	1,074 (96.58)	74 (6.89)	1,000 (93.11)	
Ever used internet	Yes	257 (23.11)	18 (7.00)	239 (93.00)	0.850
	No	855 (76.89)	57(6.69)	798(93.33)	
Wealth index combined	lowest	146 (13.13)	0 (0.00)	146 (100.00)	**0.012**
	second	155 (13.94)	14 (9.03)	141 (90.97)	
	middle	202 (18.17)	16 (7.92)	186 (92.08)	
	Fourth	304 (27.34)	21(6.91)	283 (93.09)	
	Highest	305 (27.43)	24 (7.87)	281 (92.13)	
Twins	Single	1,097 (98.65)	73 (6.65)	1,024 (93.35)	0.306
	Multiple	15 (1.35)	2 (13.33)	13 (86.67)	
Time to get water	less than 10 minutes	457 (41.10)	34 (7.44)	423 (92.56)	0.341
	10-30 minutes	227 (20.41)	13 (5.73)	214 (94.27)	
	30-60 minutes	167 (15.02)	15 (8.98)	152 (91.02)	
	More than 30 minutes	261 (23.47)	13 (4.98)	248 (95.02)	
Age of the child	0-12 months	261(23.47)	16 (6.13)	245 (93.87)	0.921
	13-24 months	185 (16.64)	15 (8.11)	170 (91.89)	
	25-36	235 (21.13)	16 (6.81)	219 (93.19)	
	37-48	217 (19.51)	13 (5.99)	204 (94.01)	
	49-60	214 (19.24)	15 (7.01)	199 (92.99)	
Type of toilet	Improved	1,060 (95.32)	75 (7.08)	985 (92.92)	**0.047**
	Un improved	52 (4.68)	0 (0.00)	52 (100.00)	
Source of drinking water	Improved	451 (40.56)	23 (5.10)	428 (94.90)	**0.071**
	Unimproved	661 (59.44)	52 (7.87)	609 (92.13)	
Household members	0-1 members	2 (0.18)	0 (0.00)	2 (100.00)	0.882
	2-3 members	163 (14.66)	9 (5.52)	154 (94.48)	
	4-5 members	400 (35.97)	27 (6.75)	373 (93.25)	
	6 or more members	547 (49.19)	39 (7.13)	508 (92.87)	

An initial investigation examined the link between maternal age and childhood diarrhea. Prevalence was 7.0% among mothers aged 20–24, the most common age group (44.96%). Younger (15–19) and older (40–44) mothers had fewer diarrhea cases in children. Although a possible difference exists between age groups, it was not statistically significant (p = 0.818). Younger mothers may still be a focus for future research due to socioeconomic factors affecting childcare and child health.

Diarrhea prevalence varied by region, with Togdheer at 12.50% and Awdal at 4.24%. This significant variation (p < 0.001) suggests environmental factors like water quality, sanitation, and healthcare access influence diarrhea. Public health interventions should be region-specific. type of residence was significantly associated with diarrhea. Nomadic families had a higher rate (8.62%) than rural (2.41%) or urban households (5.16%) (p = 0.002). Nomadic populations often face poor hygiene and water issues, indicating targeted sanitation efforts are vital. Maternal education showed a minor, non-significant difference; children of more educated mothers had a slightly higher prevalence (11.11%) vs. 6.71% in children of uneducated mothers (p = 0.677). This may reflect reporting biases or other linked factors. Diarrhea prevalence correlated with wealth. No cases were reported in the lowest quintile. The second quintile had the highest rate (9.03%) (p = 0.012), possibly due to disparities in treatment, diet, and hygiene. Wealth-related health risks need addressing. Household amenities also impacted diarrhea. No cases occurred where toilets were not upgraded. Households with upgraded toilets had a prevalence of 7.08% (p = 0.047). Improved water sources were associated with lower diarrhea rates (5.10%) compared to unimproved sources (7.87%), though not statistically significant (p = 0.071). These highlight the importance of sanitation infrastructure.

In conclusion, factors like geography, housing, wealth, and sanitation influence childhood diarrhea in Somaliland. Public health efforts should target these areas, especially vulnerable groups like nomads, to reduce diarrhea and improve child health.

### 3.2. Supervised machine learning models

Table 2 presents a comparative analysis of five supervised machine learning models: Logistic Regression, Probit Regression, Random Forest, Decision Tree, and Support Vector Machine (SVM). Each model was evaluated using key performance metrics to assess its ability to predict binary outcomes, specifically in terms of accuracy, sensitivity, specificity, positive predictive value (PPV), negative predictive value (NPV), prevalence, detection rate, balanced accuracy, and area under the curve (AUC). Understanding these metrics is crucial to determining the most suitable model for a given application.

**Table 2:** Performance metrics of supervised machine learning models for predicting diarrhea in children under five (SLDHS 2020).

	Logistic Regression		Probit Regression		Random Forest		Decision Tree		SVM	
Predicted	Yes	No	Yes	No	Yes	No	Yes	No	Yes	No
Yes	13	3	14	2	1	15	1	15	12	4
No	81	125	82	124	4	202	2	204	92	114
Accuracy	0.621		0.621		0.914		0.923		0.567	
Sensitivity	0.138		0.145		0.200		0.333		0.115	
Specificity	0.976		0.984		0.930		0.931		0.966	
Pos pred value	0.812		0.875		0.062		0.062		0.750	
Neg pred value	0.606		0.601		0.980		0.990		0.553	
Prevalence	0.423		0.432		0.022		0.013		0.468	
Detection rate	0.058		0.063		0.004		0.004		0.054	
Detection prevalence	0.072		0.072		0.072		0.072		0.072	
Balanced accuracy	0.557		0.564		0.565		0.632		0.540	
AUC	0.737		0.733		0.645		0.741		0.672	
F1 Score	0.236		0.250		0.105		0.095		0.200	

The decision tree exhibited the highest accuracy among the models, at 0.923, reflecting its capability to classify instances correctly in a transparent and interpretable manner. This model also had the highest sensitivity at 0.333, which means that it could identify one-third of the actual positive cases. This represents a significant improvement over other models, making it a better choice in scenarios where accurate detection is crucial. However, the specificity was slightly lower than that of the Random Forest (0.931) but still indicated good performance in classifying negative cases. Interestingly, while the Decision Tree exhibits favorable sensitivity, its PPV is low at 0.062, similar to that of the Random Forest, indicating a tendency to produce a high number of false positives. The low Positive Predictive Values (PPVs) of the Decision Tree and Random Forest models can be attributed to class imbalance, where the number of positive cases (children with diarrhea) was significantly lower than the number of negative instances. This imbalance can lead to models prioritizing overall accuracy over detecting positive cases. Future research should explore synthetic oversampling techniques (e.g., SMOTE) to improve PPVs.

Random Forest stands out with a much higher accuracy of 0.914, indicating that it correctly classified approximately 91.44% of the instances. This model is exceptionally robust due to its ensemble learning approach, which combines multiple decision trees to enhance predictive performance and mitigate overfitting. However, its sensitivity remained low at 0.2, indicating that it identified only 20% of the actual positives. This suggests that while Random Forest is effective in overall classification, it may miss many positive cases, which can be problematic in fields such as healthcare, where early detection is vital. The specificity of the Random Forest was 0.930, indicating that it accurately identified negative cases. Its NPV is particularly high at 0.98058, suggesting that, when it predicts a negative case, there is a 98.06% probability that it is correct. This is crucial in applications where ruling out adverse outcomes, such as quality control processes, is necessary. However, the PPV was notably low at 0.062, implying that only approximately 6.25% of the optimistic predictions were correct. This discrepancy suggests a model that, although overall accuracy may require tuning or additional feature engineering to enhance its positive prediction capabilities. An AUC of 0.645 indicates that the model has moderate discrimination ability, suggesting it may struggle to distinguish between positive and negative cases effectively.

Logistic Regression and Probit Regression yielded the same accuracy of 0.621, indicating that both models correctly classified approximately 62.16% of the instances. However, they exhibited notable differences in sensitivity and predictive capabilities. Logistic Regression has a sensitivity of 0.138, meaning that it identifies only approximately 13.83% of actual positive cases, which is relatively low. This poses a concern in scenarios where detecting positive cases is critical, such as medical diagnosis or fraud detection. Probit Regression slightly improved this metric, with a sensitivity of 0.145, allowing for marginally better identification of positive cases. Both models excel in specificity, with a Logistic Regression achieving a specificity of 0.976 and a Probit Regression at 0.984. This indicates that both models are highly effective in correctly classifying negative cases, with very few false positives. For instance, high specificity is crucial in applications such as spam detection, where misclassifying a legitimate email as spam (a false positive) can lead to significant issues.

When examining predictive values, Probit Regression showed a positive predictive value of 0.875, suggesting that when it predicts a positive outcome, there is an 87.50% chance that the prediction is correct. This high PPV can be particularly advantageous in scenarios where the cost of false positives is high, such as medical screening tests. Conversely, the negative predictive values (NPVs) for both models were moderate, with Logistic Regression at 0.606 and Probit Regression at 0.601. This indicates that when the model predicts a negative outcome approximately 60% of the time, it is accurate; however, this may not be sufficient for critical applications. The NPV is excellent at 0.99029, indicating that when it predicts a negative value, it is correct 99.03% of the time. This is particularly advantageous in scenarios where it is crucial to ensure that negative predictions are accurate and reliable. The balanced accuracy, calculated at 0.632, suggests that the model performs better across both classes than the other models. An AUC of 0.741 indicated good model discrimination, implying that it was reasonably effective at distinguishing between positive and negative outcomes.

The Support Vector Machine (SVM) demonstrated the lowest overall performance, with an accuracy of 0.567, indicating that it correctly classified just over half of the instances. This model also had the lowest sensitivity (0.115), indicating significant challenges in identifying positive cases. Such low sensitivity poses a serious concern in critical applications where missing a positive case could have dire consequences. The specificity remained relatively high at 0.966, indicating that the SVM is still effective in accurately identifying negative cases. The PPV for SVM was relatively high at 0.75, suggesting that when it predicted a positive outcome, it was correct 75% of the time. However, its NPV is lower than that of the other models at 0.553, indicating less reliable performance in predicting negative cases. An AUC of 0.672 reflects fair discrimination ability; while the model can distinguish between classes, it is not as effective as a Decision Tree or Random Forest.

Both Logistic Regression and Probit Regression demonstrate reasonable specificity but have low sensitivity, making them less suitable for scenarios requiring robust positive case identification. SVM, while showing some strengths in PPV and specificity, ultimately falls short in terms of overall performance and sensitivity, rendering it less favorable for critical applications. When selecting a model, it is essential to consider the specific needs of the application, particularly the trade-offs between false positives and false negatives. Understanding the domain context along with the implications of each metric will guide the decision-making process in choosing the most appropriate model for the task at hand.

In summary, the comparison of these five supervised machine learning models reveals distinct strengths and weaknesses across different metrics. The Decision Tree has emerged as the best overall performer in terms of accuracy and sensitivity, making it a strong candidate for applications in which identifying positive cases is critical. The Random Forest model excels in overall accuracy and NPV but struggles with sensitivity and PPV, suggesting that it may be well-suited for applications where the cost of false negatives is less critical.

### 3.3. Feature importance analysis

**Fig 1:**
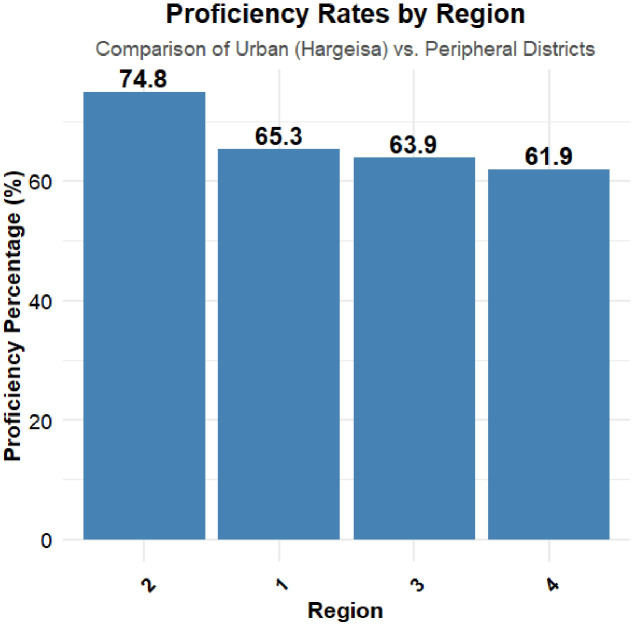
Support Vector Machine-Features Importance.

As shown Fig 1The Support Vector Machine (SVM) analysis highlights the importance of several factors, including education, Internet access, region, household television (HHtv), residence, wealth, age, household radio (HHradio), twin status, and time to water (timetowater). Education and wealth are crucial elements indicating that increased educational achievement and enhanced economic resources may be associated with positive results. Furthermore, the inclusion of media-related variables (HHtv and HHradio) suggests that access to information through these channels may affect decision-making processes and the overall results. The inclusion of demographic parameters, such as age and twin status, underscores the intricate nature of the data, suggesting that these variables may interact in various ways.

**Fig 2:**
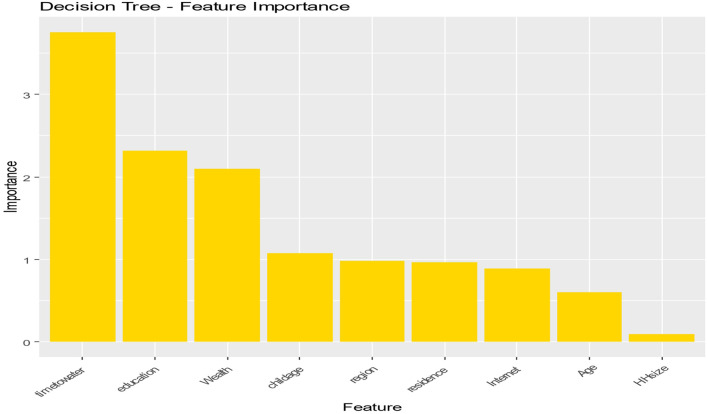
Decision Tree-Features Importance.

In the Decision Tree model (Fig 2), the rankings of feature relevance change with timetowater and education in the foremost places. This model highlights the importance of access to clean water as a key driver of outcomes, in conjunction with the fundamental impact of educational achievement. Wealth and age of children are key factors that underscore the relevance of socioeconomic position and family dynamics in the investigation. The incorporation of area and residency highlights the influence of spatial and contextual elements, indicating that living conditions significantly affect observed outcomes.

**Fig 3:**
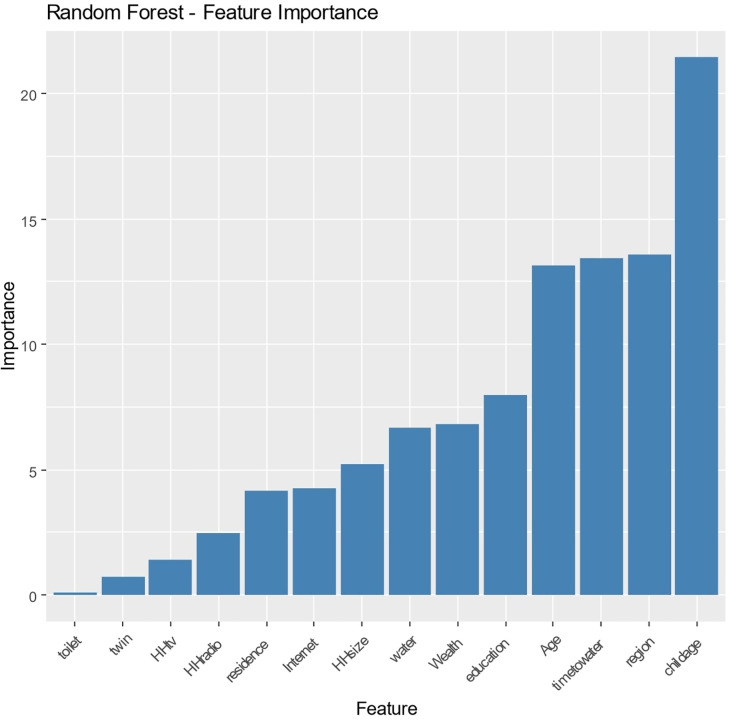
Random Forest-Features Importance.

The Random Forest model (Fig 3) enhances the comprehension of feature relevance by recognizing a diverse selection of key variables, such as wealth, child age, region, specific age cohorts, time to water, and the urban-rural dichotomy. The consistent significance of wealth and child age in several models highlights their essential roles in influencing the outcomes. The differentiation between urban and rural environments indicates a sophisticated comprehension of how geographic elements impact resource accessibility and opportunity, thereby affecting the population being examined. The diverse feature relevance of this model demonstrates the complex interaction of several factors and their combined effects on the results.

**Fig 4:**
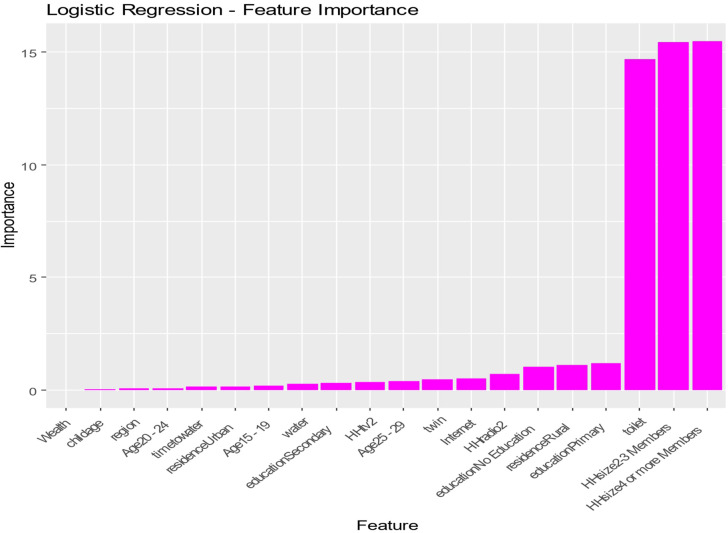
Logistic Regression-Features Importance.

The Logistic Regression model (Fig 4) confirms several findings derived from prior analyses, with wealth, child’s age, region, and education being pivotal to the investigation. The uniformity of these variables across various modeling techniques indicates strong correlations, highlighting the importance of socioeconomic and demographic components in understanding the dataset’s dynamics. Moreover, the emphasis on family size and water accessibility highlights the essential practical factors crucial for addressing the broader socio-economic challenges reflected in the data. The comparative examination of feature relevance across these varied models reveals a complex interplay of socioeconomic, demographic, and environmental factors that substantially influence the results examined. The consistent recognition of critical attributes such as wealth, education, and child age across several analytical frameworks emphasizes their significance; however, the differences in feature rankings reveal the distinct viewpoints provided by each modeling method. Thorough comprehension is essential for successfully addressing the fundamental challenges reflected in the dataset and for devising informed interventions that address these crucial drivers.

The high ranking of ‘time to water source’ and ‘type of toilet facility’ as predictors of diarrhea in our machine learning models aligns strongly with established knowledge of diarrhea risk factors. Increased time spent collecting water often reflects greater reliance on unimproved water sources and potentially unsafe storage practices, leading to a higher risk of contamination and subsequent diarrhea in children. Similarly, the type of toilet facility directly impacts sanitation and hygiene, with unimproved facilities increasing fecal contamination of the environment and promoting the transmission of diarrheal pathogens. These findings reinforce the critical role of access to safe water and adequate sanitation in preventing childhood diarrhea, consistent with numerous studies highlighting these factors as primary drivers of diarrheal disease, particularly in low-resource settings, such as Somaliland.

### 3.4. Model comparisons

**Fig 5:**
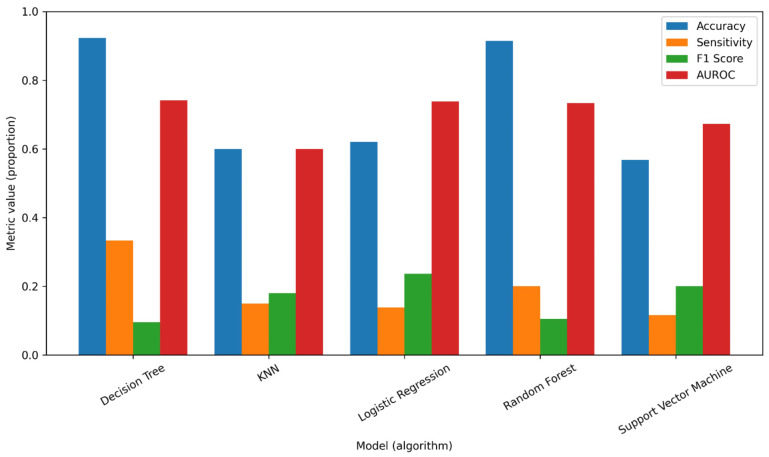
Comparison of Machine Learning Models.

The bar chart provides a comprehensive comparison of five machine learning models—Decision Tree, Logistic Regression, Probit Regression, Random Forest, and Support Vector Machine (SVM)—across four performance metrics: Accuracy, Sensitivity, F1 Score, and AUROC. These metrics are crucial for assessing the effectiveness of each model in various predictive tasks, with each metric providing insights into different aspects of model performance. See Fig 5.

Regarding the Accuracy, which reflects the overall correctness of the models in making predictions, both the Random Forest and Decision Tree models stand out with high accuracy rates, surpassing 90% (approximately 0.9144 and 0.9234, respectively). This indicates that these models perform exceptionally well in classifying instances correctly, suggesting their suitability for tasks in which the overall prediction accuracy is critical. In contrast, Logistic Regression and Probit Regression show moderate accuracy values of approximately 62%, indicating that while they perform reasonably well, they are less effective than tree-based models in terms of overall prediction correctness. The Support Vector Machine (SVM), with the lowest accuracy of approximately 56.8%, demonstrated poorer performance in correctly classifying instances, highlighting its challenges in making accurate predictions compared to the other models.

The Sensitivity metric, which measures a model’s ability to identify true positives correctly, revealed that the Decision Tree model had the highest sensitivity score of approximately 0.333. This indicates that the Decision Tree is the most effective at identifying positive cases, making it highly reliable for tasks where minimizing false negatives is paramount. The Random Forest model follows with a sensitivity of 0.2, suggesting that, while it is still effective at identifying true positives, it is not as proficient as the Decision Tree. In comparison, both Logistic Regression and Probit Regression exhibit relatively low sensitivity values of approximately 0.1383 and 0.14583, respectively, indicating that these models struggle more to identify true-positive instances. SVM had the lowest sensitivity score of 0.11538, further emphasizing its ineffectiveness in identifying true positives, which could limit its utility in applications requiring high sensitivity.

The trade-off between sensitivity and specificity in the Decision Tree and Random Forest models must be considered when interpreting results. While these models achieved high specificity, meaning they effectively classified negative cases, their low sensitivity indicates a reduced ability to detect true positive diarrhea cases. This imbalance affects the positive predictive value (PPV) and suggests that while the models are good at ruling out cases, they may miss a substantial number of children with diarrhea. Future studies should explore alternative modeling techniques, such as adjusting classification thresholds or using ensemble methods, to improve sensitivity while maintaining specificity.

In terms of the F1 Score, which balances precision and recall, Probit Regression achieved the highest score of approximately 0.25, indicating a better balance between the model’s ability to correctly classify positive cases while minimizing false positives and false negatives. Logistic Regression closely followed an F1 score of approximately 0.2364, showing a similar, though slightly less balanced, performance. SVM achieves an F1 score of 0.2, whereas Random Forest and Decision Tree exhibit notably lower F1 scores of 0.1053 and 0.0952, respectively. These lower F1 scores suggest that both the Random Forest and Decision Tree face challenges in balancing precision and recall, which could result in a higher incidence of false positives or false negatives, thus reducing their overall performance in scenarios where both precision and recall are critical.

Finally, the Area Under the Receiver Operating Characteristic Curve (AUROC) is another key metric that evaluates a model’s ability to differentiate between positive and negative classes. Here, the Decision Tree model again yields the highest AUROC value of approximately 0.7415, indicating that it is the most effective at distinguishing between the two classes, which is crucial for tasks that require robust class separation. Both Logistic Regression and Probit Regression had similar AUROC values (around 0.7374 and 0.7332, respectively), indicating moderate class separation ability, although they were not as effective as the Decision Tree. SVM and Random Forest exhibited the lowest AUROC values, with SVM at approximately 0.672 and Random Forest at 0.6459. These lower values suggest that neither model may perform as well in distinguishing between the positive and negative classes, which could limit their application in tasks requiring strong class separation.

In summary, the Decision Tree and Random Forest models demonstrated superior performance in terms of accuracy, with the Decision Tree also excelling in sensitivity and AUROC. The Decision Tree and Random Forest models were chosen due to their ability to handle complex interactions in the data, making them well-suited for predicting health outcomes. Logistic regression was included for interpretability. More complex models like Neural Networks were not employed due to concerns of overfitting and the relatively small dataset size. However, the Probit Regression model performed the best in terms of the F1 score, reflecting a more balanced trade-off between precision and recall. The SVM model, on the other hand, consistently performs poorly across all metrics, making it the least effective of the models in this comparison. These results highlight the importance of selecting a model based on the specific performance metrics that align with the task at hand, whether it is overall accuracy, sensitivity, or balanced performance, as indicated by the F1 score.

#### 3.4.1. Model comparison.

See Fig 5

## 4. Discussion

This study analyzed the determinants of diarrhea among children under five years of age in Somaliland using data from the 2020 Somaliland Demographic and Health Survey (SLDHS). The findings suggest that several factors, including time to water, education, wealth index, region, and access to sanitation facilities, significantly affect the prevalence of diarrhea among young children.

The prevalence of diarrhea varied markedly across regions, with Togdheer experiencing the highest rates. This regional disparity may reflect differences in environmental conditions, sanitation practices, and access to health care. Furthermore, children in nomadic families face a higher prevalence of diarrhea than their rural and urban counterparts, likely due to limited access to consistent sanitation facilities, clean water, and healthcare services. Additionally, the Wealth Index also showed a strong association with diarrhea prevalence, indicating that socioeconomic status plays a critical role in children’s health outcomes. Interestingly, families in the lowest wealth quintile reported no cases of diarrhea, whereas families in the higher wealth quintiles showed varying levels of prevalence. The lack of reported diarrhea cases in the lowest wealth category could be due to underreporting, as families in extreme poverty may have limited healthcare access and may not seek medical attention for mild or moderate diarrhea cases. Similar findings have been reported in studies from low-income settings [36]. improved household amenities, such as access to upgraded toilets, showed a significant relationship with a reduced prevalence of diarrhea. Households with unimproved toilet facilities had higher rates of diarrhea, underscoring the importance of sanitation in disease prevention.

Contrary to expectations, we found no significant association between maternal education and the prevalence of childhood diarrhea. This result could be influenced by reporting bias, as educated mothers may be more likely to recognize and report cases of diarrhea. Additionally, dataset limitations may have contributed to this unexpected finding. Previous studies in sub-Saharan Africa have reported mixed results regarding maternal education and child health outcomes, with some suggesting that contextual factors, including access to healthcare and sanitation infrastructure, might moderate the expected protective effect of education [37]

The analysis of machine learning models further emphasized the importance of these predictors, with Decision Tree and Random Forest models performing notably well in predicting diarrhea cases based on household and environmental factors. These models reveal intricate interactions between socioeconomic, environmental, and geographic factors that affect health outcomes. Understanding these interactions provides a basis for targeted interventions tailored to specific community needs.

The statistically insignificant relationship between maternal age and diarrhea incidence suggests the need for further exploration of the unique challenges faced by younger mothers in childcare practices. Previous studies have indicated that younger mothers often face socioeconomic difficulties that may adversely affect their children’s health [38,39].

Studies in Ethiopia [40] and Kenya [41] indicate that maternal education alone may not be a sufficient determinant of childhood diarrhea. Instead, the interplay of education with access to clean water, sanitation, and community-level health interventions often moderates the expected protective effect.

Moreover, the significant regional variations in diarrhea prevalence highlight the importance of geographical factors such as water quality and sanitation practices, which have been shown to correlate with health outcomes in similar contexts.[42,43]. The finding that Togdheer has the highest prevalence of diarrhea (12.50%) compared with Awdal (4.24%) underscores the necessity for region-specific public health interventions that address local determinants of health. [44].

The analysis also revealed that residence type significantly influenced the incidence of diarrhea, with nomadic families exhibiting a higher prevalence (8.62%) than their rural (2.41%) and urban counterparts (5.16%) [45]. This finding aligns with the literature, indicating that nomadic populations often lack consistent access to sanitation facilities, which increases their vulnerability to health issues related to poor hygiene [45]. Targeted healthcare interventions focusing on improving water and sanitation facilities for nomadic communities could be pivotal in reducing the incidence of diarrhea among children in Somaliland. The lack of association between maternal education and diarrhea may stem from reporting bias (educated mothers are more likely to report symptoms accurately) or contextual factors (e.g., a systemic lack of sanitation overriding individual education effects). The slight increase in incidence among children of more educated mothers raises questions about the interplay between education, reporting accuracy, and health-seeking behaviors [46,47]. This observation is consistent with findings from other studies suggesting that higher education levels may lead to better health literacy and awareness, potentially influencing health outcomes [48].

Economic factors, particularly the wealth index, have emerged as a significant determinant of diarrhea prevalence, with households in the lowest income quintile reporting no cases of diarrhea [44,49]. This finding reflects the broader literature that links poverty to inadequate access to sanitation and healthcare services, thereby increasing susceptibility to infections, such as diarrhea [50]. The correlation between household amenities, such as access to improved toilets and water sources, further emphasizes the critical role of sanitation in preventing diarrhea [51]. These data suggest that enhancing water and sanitation infrastructure should be a priority for public health initiatives aimed at reducing the incidence of diarrhea.

The machine learning models employed in this study provide a robust framework for predicting the incidence of diarrhea based on various factors. The Decision Tree model, with its highest accuracy and sensitivity, has emerged as the most effective tool for identifying positive cases, which is crucial in public health settings where early detection can significantly impact health outcomes. The Random Forest model exhibits high overall accuracy and struggles with sensitivity, indicating the need for further refinement to enhance its predictive capability. Policymakers should utilize data-driven approaches, such as insights from Decision Tree models to direct sanitation improvements toward high-risk areas, like Togdheer. Efforts should focus on cost-effective interventions, including increasing access to latrines and promoting the use of household water purification techniques.

In conclusion, the findings from the SLDHS underscore the multifaceted nature of diarrhea prevalence among children in Somaliland, highlighting the need for tailored public health interventions that consider regional, socioeconomic, and environmental factors. Interventions such as the Community-Led Total Sanitation (CLTS) initiative, which has been successfully implemented in Ethiopia and Kenya, have resulted in significant reductions in childhood diarrhea cases. Additionally, the distribution of water purification tablets in rural areas and hygiene education campaigns targeting caregivers have been effective in improving household sanitation practices and reducing disease transmission

## 5. Limitation

The low sensitivity (e.g., 33.33% in the Decision Tree model) reflects class imbalance, given the low prevalence of diarrhea (7.2%) in the dataset. Future studies should employ techniques such as the Synthetic Minority Over-sampling Technique (SMOTE) to balance class distribution and improve model sensitivity.

## 6. Future Work

Future research should address the limitations of the current study and explore additional factors affecting childhood diarrhea in Somaliland. First, a longitudinal study design would be beneficial for assessing changes in the prevalence of diarrhea and its determinants over time. This study is subject to recall bias, as mothers may not accurately recall episodes of diarrhea. Additionally, measurement errors in survey data collection and the cross-sectional nature of the study prevent us from drawing causal inferences.

Furthermore, more comprehensive data collection on environmental factors, particularly water quality and seasonal variations, could refine our understanding of how these variables influence the prevalence of diarrhea. An in-depth analysis of the impact of maternal health knowledge and childcare practices on child health outcomes is also recommended, given the surprising findings regarding maternal education. Additionally, exploring community-based interventions and assessing their efficacy across different regions and residence types may offer guidance in tailoring public health initiatives.

Finally, applying machine learning methods with a broader range of models and incorporating additional predictive variables, such as household nutrition and immunization status, could improve predictive accuracy and enhance our understanding of the determinants of childhood diarrhea in Somaliland. Future studies should incorporate geospatial analysis to map high-risk areas and gain a better understanding of the geographic distribution of diarrhea prevalence.
